# Cost-effectiveness and budget impact of the management of uncomplicated urinary tract infection by community pharmacists

**DOI:** 10.1186/s12913-019-4303-y

**Published:** 2019-07-18

**Authors:** Chiranjeev Sanyal, Donald R. Husereau, Nathan P. Beahm, Daniel Smyth, Ross T. Tsuyuki

**Affiliations:** 1 Canadian Pharmacists Association, 1785 Alta Vista Drive, Ottawa, Ontario K1G 3Y6 Canada; 2 School of Epidemiology and Public Health, University of Ottawa, 600 Peter Morand Crescent, Ottawa, Ontario K1G 5Z3 Canada; 3 Faculty of Pharmacy and Pharmaceutical Sciences, University of Alberta, 3-213 Edmonton Clinic Health Academy, 11405 87 Avenue NW, Edmonton, Alberta T6G 1C9 Canada; 4 Division of Infectious Diseases, Department of Medicine The Moncton Hospital, Dalhousie University, 135 MacBeath Ave, Moncton, New Brunswick E1C 6Z8 Canada; 5 EPICORE Centre, Department of Pharmacology, Faculty of Medicine and Dentistry, University of Alberta, 9-70B Medical Sciences Building, Edmonton, Alberta T6G 2B7 Canada

**Keywords:** Cost-effectiveness, Budget impact, Urinary tract infection, Pharmacist services

## Abstract

**Background:**

Urinary tract infections (UTI) are one of the most common infections treated in primary care and the emergency department. The R_x_OUTMAP study demonstrated that management of uncomplicated UTI by community pharmacists resulted in high clinical cure rates similar to those reported in the literature and a high degree of patient satisfaction. The objective of this study was to assess the cost-effectiveness and budget impact of community pharmacist-initiated compared to family or emergency physician-initiated management of uncomplicated UTI.

**Methods:**

A decision analytic model was used to compare costs and outcomes of community pharmacist-initiated management of uncomplicated UTI to family or emergency physician-initiated management. Cure rates and utilities were derived from published studies. Costs of antibiotic treatment and health services use were calculated based on cost data from Canada. We used a probabilistic analysis to evaluate the impact of treatment strategies on costs and quality-adjusted-life-months (QALMs). In addition, a budget impact analysis was conducted to evaluate the financial impact of community pharmacist-initiated uncomplicated UTI management in this target population. This study was conducted from the perspective of the public health care system of Canada.

**Results:**

Pharmacist-initiated management was lower cost ($72.47) when compared to family and emergency physician-initiated management, $141.53 and $368.16, respectively. The QALMs gained were comparable across the management strategies. If even only 25% of Canadians with uncomplicated UTI were managed by community pharmacists over the next 5 years, the resulting net total savings was estimated at $51 million.

**Conclusion:**

From a Canadian public health care system perspective, community pharmacist-initiated management would likely be a cost-effective strategy for uncomplicated UTI. In an era of limited health care resources, expanded roles of community pharmacists or other non-physician community based prescribers are important mechanisms through which accessible, high-quality and cost-effective care may be achieved. Further studies to evaluate other conditions which can be managed in the community and their cost effectiveness are essential.

## Background

Urinary tract infection (UTI) is one of the most common bacterial infections seen in primary care affecting about 12–15% of women annually and 50% of women by 32 years of age. [1, 2] It is associated with lower abdominal pain, discomfort, and stress that can significantly affect quality-of-life. In Canada, UTI is the eighth and fifth most common reasons for ambulatory clinic and emergency department visits, respectively [1, 2]. UTI is a common condition leading to initiation of antibiotic treatment [1, 2]. The annual cost of treating UTIs is substantial, with studies from France and the US reporting €58 million and $2.47 billion, respectively [3, 4]. 

The emerging paradigm of patient-centered care emphasizes patient preference and values and puts patients’ interests at the center of the decision-making process to ensure patients have timely access to high quality care and greater choice in how, where, and when to access services [5, 6]. Community pharmacies provide an opportunity for patients to access a regulated health care professional (i.e., pharmacists) without an appointment to seek treatment for uncomplicated UTI among other conditions. This enables pharmacists to assess patients, implement appropriate antibiotics (if necessary), reduce the burden on other areas of the health care system, help patients access care during or after working hours, and provide economically efficient delivery of health care.

Health care decision makers (payers) are increasingly seeking evidence to decide upon the value of a service to be recommended for public funding. Value-based care aims to maximize the health of the population per dollar invested. Both patient-centered and value-based care aim to produce outcomes that will benefit patients and the health care system. To date, there is a lack of studies that have evaluated the economic benefit of the management of uncomplicated UTI by community pharmacists compared to family or emergency physicians.

The RxOUTMAP study [2] demonstrated a high rate of clinical cure, high rate of guideline concordance, and high patient satisfaction with pharmacist prescribing and care for uncomplicated UTIs. Therefore, the objective of this study was to conduct a cost-effectiveness analysis of the role of community pharmacists compared to family or emergency physicians in the management of uncomplicated UTI, from the perspective of the public health care system of Canada. A budget impact analysis was also conducted to estimate the impact of adopting community pharmacist-initiated management of uncomplicated UTI on the Canadian public health care system over a period of 5 years.

## Methods

We followed the Consolidated Health Economic Evaluation Reporting Standards (CHEERS) statement [7] to report the details of this study. We conducted an economic evaluation in the form of a cost-effectiveness analysis, of the R_x_OUTMAP study, a prospective study of community pharmacist prescribing and care for women presenting with uncomplicated UTI in New Brunswick between June 2017 to April 2018. [2] The details of this study can be found elsewhere [2]. Briefly, women presented to community pharmacists with symptoms suggestive of uncomplicated UTI. Upon consultation with the pharmacist, they were assessed for UTI and prescribed antibiotics if necessary, guided by recently published guidelines [1] (this was called the pharmacist-initiated arm). In patients who were ascertained to have uncomplicated UTI by a physician and who were prescribed antibiotics (the physician-initiated arm), pharmacists assessed the patient, reviewed their prescription, and may have performed prescription adaptation or therapeutic substitution, as needed [2].

For the pharmacist-initiated group, the proportion of patients who received initial treatment with nitrofurantoin [100 mg twice daily for 5 days], trimethoprim/sulfamethoxazole (TMP-SMX) [160/800 mg twice daily for 3 days], fosfomycin [3 g single dose], cefuroxime [500 mg twice daily for 7 days], and fluoroquinolones [norfloxacin - 400 mg twice daily for 4 days or ciprofloxacin 500 mg twice daily for 4 days] was 88, 8, 2, 1, and 1%, respectively [8].

In the physician-initiated group, the distribution of initial treatment prescribed by the physicians with nitrofurantoin [100 mg twice daily for 7 days], TMP-SMX [160/800 mg twice daily for 5 days], fosfomycin [3 g single dose], cefuroxime [500 mg twice daily for 7 days], fluoroquinolones [norfloxacin - 400 mg twice daily for 6 days or ciprofloxacin 500 mg twice daily for 6 days], and TMP [100 mg twice daily for 5 days] was 55, 26, 4, 2, 11, and 2%, respectively [8]. 

### Target population

Our population of interest was adult women with symptoms indicating UTI and assessed to be uncomplicated by pharmacists, or those presenting with a prescription for antibiotics from family physicians who were assessed to have uncomplicated UTI. In addition, we considered patients who present to the emergency department with symptoms indicating UTI assessed to be uncomplicated and received a prescription for an antibiotic from emergency physicians.

### Setting and location

This study represents a real-world clinical setting in New Brunswick, Canada where women with symptoms of uncomplicated UTI (e.g., dysuria, frequency) are usually managed either by family physicians, or emergency physicians in primary care. However, with expanded scope of practice, community pharmacists are uniquely positioned to manage uncomplicated UTI.

### Study perspective

We conducted this study from the perspective of the public health care system of Canada for the reference case. Therefore, costs and outcomes were considered from the viewpoint of the publicly funded health care payer.

### Interventions

Three care pathways were evaluated: (i) community pharmacist-initiated management – a novel strategy where community pharmacists examined patients with symptoms of uncomplicated UTI and initiated antibiotic treatment (community pharmacist-initiated), (ii) family physician-initiated management – wherein family physicians examined patients with symptoms of uncomplicated UTI and initiated antibiotic treatment (family physician-initiated), and (iii) emergency physician-initiated management – patients sought care at the emergency department (ED) and the antibiotic treatment was initiated by an emergency physician (emergency physician-initiated). We included the emergency physician management strategy because patients with uncomplicated UTI might choose to go to the emergency department to seek care, but the costs associated with this decision could be significantly different than seeing a family physician at their clinic.

### Time horizon

We considered a fourteen day follow-up for the initial treatment [2]. After initial treatment, patients who did not achieve resolution of symptoms received a second round of treatment and were followed for an additional sixteen days. Overall, the time horizon of the study was one month. Following these treatment courses, all patients were assumed to achieve resolution of symptoms.

### Discount rate

Costs and outcomes were not discounted in our model because the time horizon was less than one year.

### Clinical outcomes and effectiveness

Clinical cure was measured as the percentage of women who have achieved resolution of symptoms of uncomplicated UTI with the antibiotics prescribed. [2] There is a lack of studies that have evaluated the management of uncomplicated UTI by community pharmacists compared to family or emergency physicians, especially in the Canadian context. Therefore, we developed the model based on the data reported by the RxOUTMAP study [2].

### Health outcomes

Utilities are weights that represent preference for certain health outcomes. A higher utility indicates more preferable health outcome and improved health-related-quality-of-life. Utilities are measured on a scale of 0 (death) to 1 (full health) [9]. Uncomplicated UTI is an acute infection; therefore, health outcomes were quantified as quality-adjusted-life-months (QALMs). An area under the curve method was used to estimate QALMs for the strategies evaluated. The changes in the QALMs, a product of a life-month multiplied by health state preference values (utilities) for different states of health, between the strategies was estimated as the difference in the area under the curve over the time horizon of the study. [10] As all patients were expected to stay alive for one month, the QALMs assigned to individual interventions are dependent on the probability of achieving resolution of infection. We used utility values from Ernst et al., because they reported utilities that correspond to our model structure, the study population and choice of antibiotics was comparable to our study. [10]

### Resource use and costs

Direct costs from the perspective of the public health care system of Canada were considered for modeling, which included pharmacist visits, family or emergency physician visits, and treatment with antibiotics for initial and subsequent therapy. All costs are presented in $CAD 2018 (Table 1). Wherever necessary, costs were adjusted for inflation using the Statistics Canada, Consumer Price Index [11] .Unit costs of antibiotics were obtained from the New Brunswick prescription drug program [12]. The costs of family or emergency physician visits were derived from Canadian data sources [13, 14]. The cost of pharmacist assessments was based on expert opinion.

**Table 1:** Model input parameters

Parameter	Value	References
Cure rates		
*Pharmacist-initiated arm*	88.6%	[2]
*Family physician-initiated arm*	90%	[15]
*Emergency physician-initiated arm*	90%	[15]
Persistent infection		
*Community pharmacist-initiated arm*	11.4%	[2]
*Family physician-initiated arm*	10.0%	Expert Opinion, [15]
*Emergency physician-initiated arm*	10.0%	Expert Opinion, [15]
Health care professional costs		
*Community pharmacist visit*	$23.00	Expert opinion
*Family physician initial visit*	$77.20	[13]
*Family physician follow-up visit*	$38.35	[13]
*Emergency physician/department visit*	$304.8	[13, 14]
Medication costs, (initial treatment)		
*Community pharmacist-initiated arm*	$14.47	Calculated, [12, 16]
*Family physician-initiated arm*	$16.90	Calculated, [12, 16]
*Emergency physician-initiated arm*	$16.90	Calculated, [12, 16]
Medication costs, (subsequent treatment)		
*Extended antibiotic treatment by community pharmacists*	$14.47	Calculated, [12, 16]
*Extended antibiotic treatment by family physicians*	$16.90	Calculated, [12, 16]
*Antibiotic switch to fluoroquinolones*	$18.47	Calculated, [12, 16]
Utility		
Baseline	0.68	[10]
Cured following initial treatment	0.83	[10]
Persisted following initial treatment	0.76	[10]
Cured following subsequent treatment	0.82	Assumption, [10]

### Model structure

#### Reference case

**Fig. 1:**
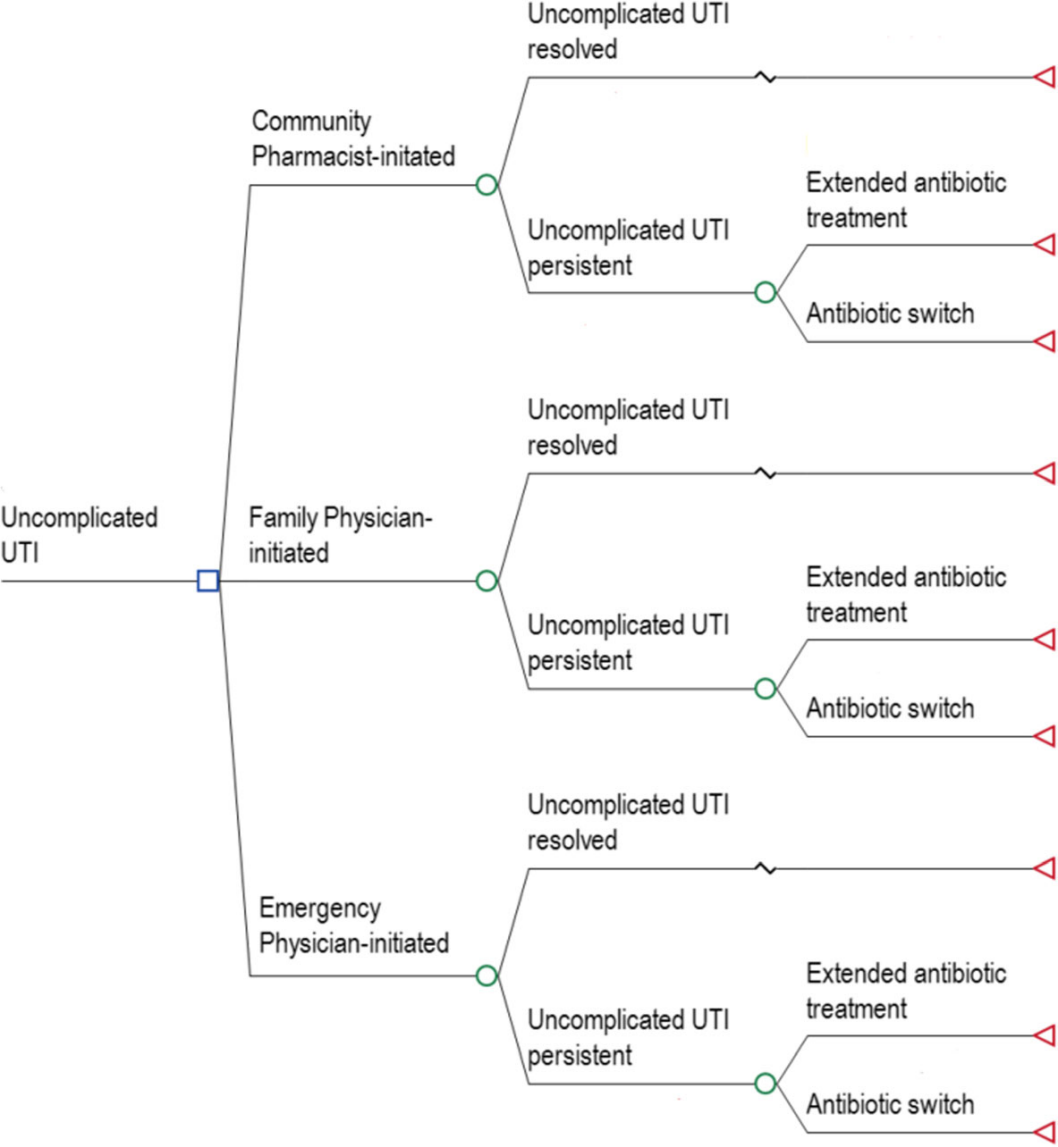
Decision tree model for cost-effectiveness analysis of uncomplicated UTI

A decision analytic model (Fig. 1) was developed to determine the incremental cost per QALM gained from the community pharmacist-initiated arm compared to family or emergency physician arm.

In the model, the initial decision node represents the patient’s choice of management strategy (Fig. 1). Each strategy is incorporated as a distinct branch following the decision node, representing the sequence of events a patient may experience within a strategy. In the model, patients were prescribed an antibiotic treatment regimen by community pharmacists, family physicians, or emergency physicians. Following initial treatment, the symptoms of uncomplicated UTI were either resolved or persisted. In the case of persistent UTI, patients either received extended treatment with the antibiotic prescribed initially (if they were non-adherent) or switched to a fluoroquinolone.

#### Model assumptions

The following assumptions were made to develop the model:

- Treatment distribution for family or emergency physicians was the same.
- Resolution rates of uncomplicated UTI for patients treated by family or emergency physicians was the same.
- Persistent uncomplicated UTI (following initial treatment) was resolved over one month of follow-up.
- None of the patients experienced adverse events that required medical attention.
- Utilities were independent of type of management strategy.

### Analyses

#### Costing

At each stage of the model, service use such as pharmacist visit, family or emergency physician visit, and antibiotic use was recorded by the model. Each strategy in the model was costed by applying unit costs (Table 1) to the expected service volumes associated with that strategy.

This study was conducted from the perspective of the public health care system; therefore, resource use that falls out of public reimbursement were not considered for reference-case costing.

#### Health outcomes

The impact of uncomplicated UTI and its treatment on the quality of life of women was estimated for each of the strategies. The health benefit was estimated as the QALMs gained for each of the strategies.

#### Cost-effectiveness

We used a cost-utility analysis, a type of economic evaluation that measures costs and impact of different management strategies on quality-of-life. The costs and QALMs associated with each strategy through the decision tree were used in conjunction with the pathway probabilities to estimate the mean expected costs and QALMs. There were more than two interventions being compared to the novel strategy (pharmacist-initiated); therefore, the expected costs and QALMs of the comparators and the corresponding incremental cost-effectiveness ratio (ICER) were calculated sequentially. [9]

The parameters used in the model are associated with some uncertainty. We conducted a probabilistic analysis by drawing values from distributions reflecting the underlying uncertainty of parameters. We performed 10,000 Monte Carlo simulations to estimate the average costs and QALMs for each of the management strategies. Then, we determined the ICER using sequential analysis which compared all the strategies and ranked them by increasing cost [9] .If a strategy was dominated (i.e., more costly and less effective than at least one other strategy) or was subject to extended dominance (i.e., ICER is higher than the next most effective strategy), it was removed from the analysis, and all remaining strategies were re-estimated until only non-dominated strategies were identified [9].

Model input parameters with uncertainty were varied according to pre-defined distributions simultaneously ±25% of the point estimates summarized in Table 1. We applied beta distribution to probabilities and utilities, and gamma distribution to cost parameters [9]. Uncertainty around model parameters was estimated as a 95% CI.

#### Sensitivity and scenario analyses

We examined the sensitivity of the results to underlying assumptions regarding cure rates. For the reference case, the cure rate of community pharmacist-initiated management was based on a single study; [2] therefore, we examined how changes in cure rates would influence the reference case findings. We examined scenarios where cure rates associated with the community pharmacist-initiated management was varied to 86 and 83%.

We also used a broader cost perspective to conduct a scenario analyses from a societal perspective by accounting for productivity losses and out-of-pocket costs that could be incurred to attend an appointment at the family physician’s clinic or to visit the emergency department.

For out-of-pocket costs, we considered costs borne by patients to park their vehicle at the hospital or take public transport. We assumed the number of hours a patient will spend on average at the emergency department and multiplied this by a daily parking rate of $1.75/h [17]. Alternatively, patients may take public transport which will cost them $2.75 each way [18].

We calculated productivity loss in dollars for adults who had to visit the family practitioner’s clinic or the emergency department to examine their symptoms and receive an antibiotic prescription. To see a family or emergency physician, we assumed patients will spend 4 h or 8 h, respectively. We calculated lost productivity by multiplying the number of hours a patient will miss from work by the average hourly wage of $22.97 in New Brunswick [19].

We assumed patients seeking care from community pharmacists will not have to take time off work. However, patients taking public transport will pay for their bus fare.

All scenario analyses were conducted using a probabilistic model. We applied beta distribution to probabilities and utilities, and gamma distribution to cost parameters [9]. 

#### Budget impact analysis

A budget impact analysis (BIA) was conducted to examine the changes in budget to the Canadian public health care system considering an increased uptake of community pharmacist-initiated management. We estimated the net budget impact using the cost difference between two scenarios: family and emergency physician-initiated management (the current scenario) and the anticipated practice of community pharmacist-initiated management (the new scenario).

To estimate our target population, based on the literature, we assumed 12% of women annually would seek treatment for uncomplicated UTI [2, 20]. The target population was women in Canada aged 25 years or older with symptoms of uncomplicated UTI. Assuming a population growth of 1% per year and 12% of women would annually seek care for uncomplicated UTI, we estimated approximately 1,614,384 and 1,679,934 women would seek care in year 1 and year 5, respectively.

Currently, there is a lack of public funding for community pharmacist-initiated antibiotic treatment for the management of uncomplicated UTI. Therefore, based on expert consultation, we assumed the current uptake rate to be 1% annually. For the reference case analysis, based on expert consultation, we assumed the uptake rate would increase to 25% over the next five years. The net budget impact was estimated as the cost difference between the new scenario and the current scenario. For scenario analyses, we increased the future uptake rate community pharmacist-initiated management to 50 and 75% over the next five years.

## Results

### Cost-effectiveness of management strategies

#### Reference case analysis

**Fig. 2:**
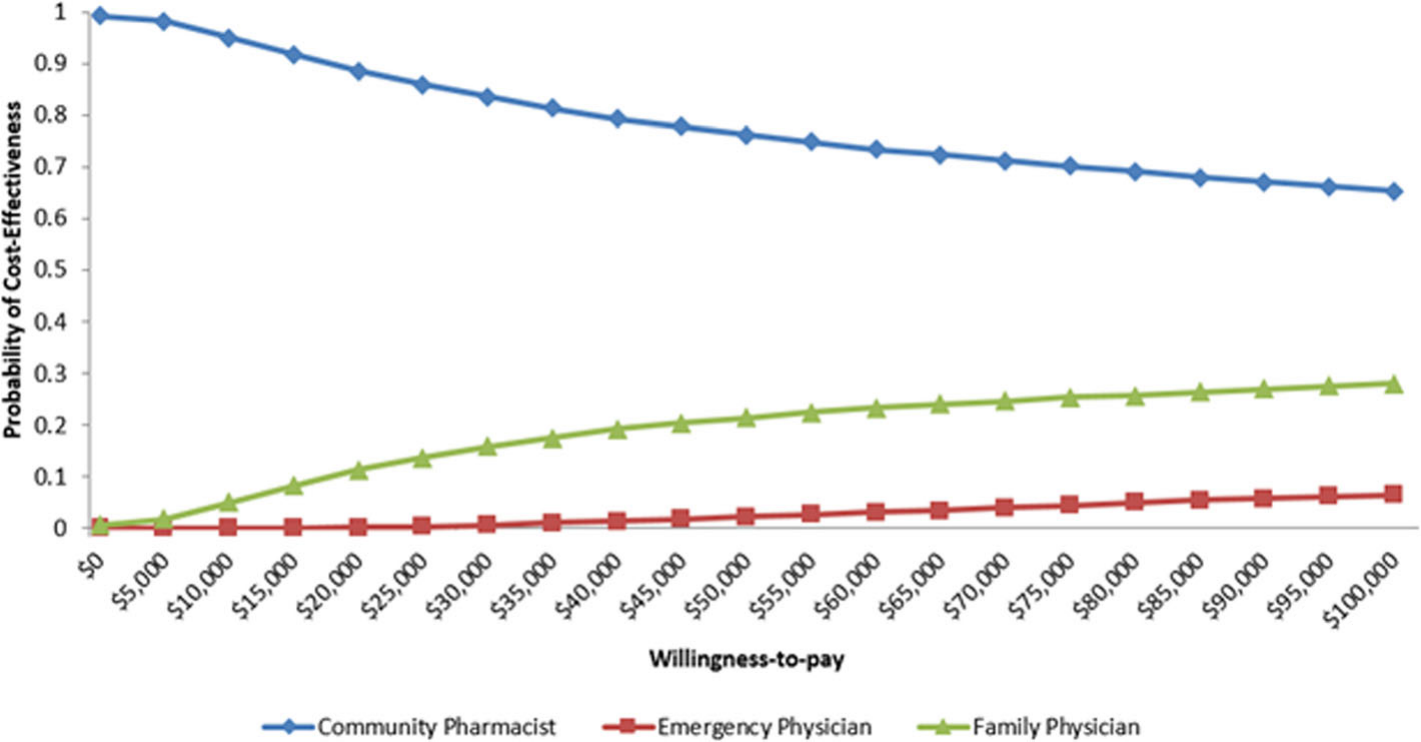
Cost-effectiveness Acceptability Curve – community pharmacist-initiated, family physician-initiated, and emergency physician-initiated management of uncomplicated UTI

The sequential analysis for our reference case indicated two non-dominated strategies: community pharmacist- and family physician-initiated management (Table 2). The emergency physician-initiated management was subject to extended dominance. The mean costs of community pharmacist-initiated, family physician- and emergency physician-initiated management were $72.47, $141.53, and $368.16, respectively (Table 2). The mean QALMs of community pharmacist-initiated, family physician and emergency physician-initiated management were 0.75137, 0.75142, and 0.75146, respectively (Table 2). The CEAC showed different WTP thresholds affected the probability of cost-effectiveness of the management strategies (Fig. 2).

**Table 2:** Cost-effectiveness, Reference-case

Strategy	Mean Costs, $	Mean Effects, QALMs	Incremental Costs, $	Incremental Effects, QALMs	ICER^†^, $/QALM gained
Community pharmacist-initiated	72.47 (49.74; 99.61)	0.75137 (0.50954; 0.92395)	–	–	–
Family physician-initiated	141.53 (96.11; 198.26)	0.75142 (0.50910; 0.92384)	69.06 (16.16; 129.67)	0.00005 (−0.00673; 0.00718)	1,381,200
Emergency physician-initiated	368.16 (227.21; 543.26)	0.75146 (0.51983; 0.92400)	–	–	Extended dominance

^†^Sequential analysis

### Sensitivity and scenario analyses

#### Sensitivity to cure rates

The ICER of family physician-initiated versus community pharmacist-initiated management was sensitive to alternate assumptions regarding cure rates following initial treatment (Table 3). Moreover, the mean costs of the management strategies were modestly higher due to the larger number of persistent uncomplicated UTI cases requiring subsequent treatment, compared to the reference case. Similar to the reference case, emergency physician-initiated management was subject to extended dominance.

**Table 3:** Cost-effectiveness, Sensitivity Analysis

Strategy	Mean Costs, $	Mean Effects, QALMs	Incremental Costs, $	Incremental Effects, QALMs	ICER^†^, $/QALM gained
*Cure rate of community pharmacist-initiated antibiotic-treatment (86%)*					
Community pharmacist-initiated	72.81 (49.77; 99.77)	0.75229 (0.51536; 0.92437)	–	–	–
Family physician-initiated	142.01 (95.73; 197.40)	0.75233 (0.51488; 0.92407)	69.20 (15.50; 129.40)	0.00004 (−0.00726; 0.00698)	1,730,000
Emergency physician-initiated	371.09 (232.14; 546.54)	0.75237 (0.51973; 0.92503)	–	–	Extended dominance
*Cure rate of community pharmacist-initiated antibiotic-treatment (83%)*					
Community pharmacist-initiated	72.90 (50.07; 100.88)	0.75326 (0.52212; 0.92515)	–	–	–
Family physician-initiated	141.74 (94.71; 197.46)	0.75328 (0.52284; 0.92450)	68.85 (14.61; 129.57)	0.00002 (−0.00714; 0.00701)	3,442,500
Emergency physician-initiated	370.36 (229.47; 546.86)	0.75333 (0.52349; 0.92436)	–	–	Extended dominance

^†^Sequential analysis

#### Societal perspective

The mean costs of community pharmacist-initiated, family physician- and emergency physician-initiated management were $78.70, $239.43, and $609.78, respectively, with a mean of 0.75232, 0.75236, and 0.75239 QALMs, respectively (Table 4). From the societal perspective, the mean cost of each of the treatment strategies was more (Table 4) compared to the reference case. This was due to the additional costs borne by patients due to transportation and/or time off from work to see their health care professional. Moreover, from a societal perspective, emergency physician-initiated was subject to extended dominance.

**Table 4:** Cost-effectiveness, Societal Perspective

Strategy	Mean Costs, $	Mean Effects, QALMs	Incremental Costs, $	Incremental Effects, QALMs	ICER^†^, $/QALM gained
Community pharmacist-initiated	78.70 (54.27; 108.25)	0.75232 (0.51506; 0.92437)	–	–	–
Family physician-initiated	239.43 (162.36; 327.39)	0.75236 (0.51555; 0.92461)	161.09 (80.29; 255.07)	0.00004 (−0.00685; 0.00709)	4,027,250
Emergency physician-initiated	609.78 (382.65; 894.90)	0.75239 (0.51676; 0.93418)	–	–	Extended dominance

^†^Sequential analysis

### Budget impact of management strategies

#### Reference case

In the current scenario there is a lack of public funding and the scope of practice limits access to community pharmacist-initiated antibiotic treatment. In contrast, expanding its access to 25% of the target population Canada-wide through public funding would lead to a net savings estimated at $2.9 million in year 1 and $16.3 million in year 5, respectively (Table 5). The total net savings over 5 years was estimated at $51.1 million (Table 5).

**Table 5:** Budget Impact, Reference Case - Expanding community pharmacist-initiated management to 25% of target population

Total Cost per Year, $						
Strategy	Year 1	Year 2	Year 3	Year 4	Year 5	5-Year Total
Current Scenario	242,003,570	244,423,605	246,867,841	249,336,520	251,829,885	1,234,461,421
New Scenario	239,059,337	237,913,234	235,422,846	235,479,723	235,514,195	1,183,389,335
Net Budget Impact^†^	-2,944,233	−6,510,371	−11,444,995	−13,856,797	−16,315,690	−51,072,086

^†^Negative results indicate cost savings

#### Scenario analyses

Table 6 and Table 7 showed significant savings with increased public funding Canada-wide for community pharmacist-initiated management to provide care to 50 and 75% of the target population. The net budget impact of 50% of the target population receiving community pharmacist-initiated management would lead to a net savings estimated at $6.7 million to $37.3 million in year 1 and year 5, respectively (Table 6). The total net savings over 5 years was estimated at $102.7 million (Table 6). Similarly, 75% of the target population receiving community pharmacist-initiated management would result in net savings estimated at $9.5 million to $48.4 million in year 1 and year 5, respectively (Table 7). The total net savings over 5 years was estimated at $151.7 million (Table 7).

**Table 6:** Budget Impact, Scenario Analysis - Expanding community pharmacist-initiated management to 50% of target population

Total Cost per Year, $						
Strategy	Year 1	Year 2	Year 3	Year 4	Year 5	5-Year Total
Current Scenario	284,721,145	287,568,357	290,444,040	293,348,481	296,281,966	1,452,363,989
New Scenario	277,977,982	274,423,011	271,626,595	266,719,655	258,957,513	1,349,704,756
Net Budget Impact^†^	−6,743,164	−13,145,346	−18,817,445	−26,628,826	−37,324,453	−102,659,233

^†^Negative results indicate cost savings

**Table 7:** Budget Impact, Scenario Analysis - Expanding community pharmacist-initiated management to 75% of target population

Total Cost per Year, $						
Strategy	Year 1	Year 2	Year 3	Year 4	Year 5	5-Year Total
Current Scenario	284,721,145	287,568,357	290,444,040	293,348,481	296,281,966	1,452,363,989
New Scenario	275,169,889	266,950,012	258,538,221	252,148,961	247,845,699	1,300,652,782
Net Budget Impact^†^	−9,551,256	−20,618,345	−31,905,820	−41,199,520	−48,436,267	−151,711,207

^†^Negative results indicate cost savings

## Discussion

To our knowledge, this is the first study to report the cost-effectiveness and budget impact of community pharmacist-initiated management of uncomplicated UTI compared to family or emergency physician-initiated. Our model results showed emergency physician-initiated management was subject to extended dominance (i.e., ICER is higher than the next most effective strategy) in all analyses. Of the remaining two non-dominated strategies, community pharmacist-initiated management was less costly and gave comparable QALMs compared to family physician-initiated. The corresponding ICER comparing family physician-initiated to community pharmacist-initiated management was approximately $1.4 million per QALM gained.

Similar to a previous study, [21] we have used QALM as our outcome measure as it is more plausible for acute conditions compared to quality-adjusted-life-years (QALYs), which is more appropriate for chronic conditions. Both for the reference case and scenario analyses, the estimated ICERs of family physician-initiated compared to community pharmacists was higher than commonly used willingness-to-pay thresholds. These high ICERs resulted from modest differences in QALMs. These modest differences in QALMs between management strategies resulted from uncomplicated UTI being an acute condition. In addition, the relative cure rates following initial treatment were similar among management strategies. Due to the modest incremental QALM, the lowest cost strategy, community pharmacist-initiated management, likely gives good value for money.

The results from a societal perspective (scenario analysis) compared to the public health care system (reference case) indicate the relatively high cost associated with all management strategies. This is due to the cost associated with lost productivity and/or transportation fares. Currently, if family physicians are not available to provide care after-hours or during the weekend, the patient would need to decide between either waiting until a physician is available again, or utilize the far more expensive option of seeing an emergency physician. However, patients could have the option of accessing a community pharmacist in this setting, instead. Therefore, pharmacist-initiated management of uncomplicated UTI has tremendous potential to provide economically viable patient-centered care. The findings of the cost-effectiveness analysis are generaliizable to specific patient populations addressed in the study we investigated.

The BIA provided important information to decision makers who need to know the financial consequences of adopting community pharmacist-initiated UTI management in routine practice. Our results showed public funding of community pharmacist-initiated management would lead to significant savings to the health care system.

There are limitations to our study. First, evidence for the clinical effectiveness of community pharmacist-initiated treatment was based on a single prospective study without a comparator group. [2] Nevertheless, the clinical cure rates were similar to those described in the medical literature. [16] Second, the drug costs included in our analyses reflect the province of New Brunswick, which may differ from other provinces in Canada. Third, utility weights used in our analysis were independent of health care provider.

## Conclusions

The value and affordability of services delivered by community pharmacists practicing to their full scope needs to be evaluated to help public payors decide upon recommending it for funding and implementation. In the case of management of uncomplicated UTI by community pharmacists, significant savings in healthcare expenditure would be realized. Moreover, providing patients with access to this full scope of pharmacy services for uncomplicated UTI Canada-wide would save millions of dollars. Additionally, the RxOUTMAP study showed that patients preferred treatment by their pharmacist. These study findings present a compelling argument to implement these changes.

## Data Availability

All data generated or analysed during this study are included in this published article.

## Abbreviations

BIA: Budget impact analysis
CAD: Canadian Dollar
CHEERS: Consolidated Health Economic Evaluation Reporting Standards
ED: Emergency department
ICER: Incremental cost-effectiveness ratio
QALMs: Quality-adjusted-life-months
QALYs: Quality-adjusted-life-years
RxOUTMAP: Outcomes of Urinary Tract Infection Management by Pharmacists
UTI: Urinary tract infections
WTP: willingness-to-pay
